# Colonization of Methicillin-Resistant *Staphylococcus aureus* (MRSA) among Medical Students in Tertiary Institution in Central Malaysia

**DOI:** 10.3390/antibiotics9070382

**Published:** 2020-07-06

**Authors:** Sanjiv Rampal, Nur Hidayah Zainuddin, Nur Athirah Elias, Tengku Zetty Maztura Tengku Jamaluddin, Sandra Maniam, Seoh Wei Teh, Suresh Kumar Subbiah

**Affiliations:** 1 Department of Orthopedic and Traumatology, Faculty of Medicine & Health Sciences, Universiti Putra Malaysia, Serdang 43400, Selangor Darul Ehsan, Malaysia; 2 Medical Programme, Faculty of Medicine & Health Sciences, Universiti Putra Malaysia, Serdang 43400, Selangor Darul Ehsan, Malaysia; hz9.two@gmail.com (N.H.Z.); nurathirahelias@gmail.com (N.A.E.); 3 Department of Medical Microbiology and Parasitology, Faculty of Medicine & Health Sciences, Universiti Putra Malaysia, Serdang 43400, Selangor Darul Ehsan, Malaysia; tengkuzetty@upm.edu.my (T.Z.M.T.J.); seohwei1208@gmail.com (S.W.T.); suresh@upm.edu.my (S.K.S.); 4 Department of Human Anatomy, Faculty of Medicine & Health Sciences, Universiti Putra Malaysia, Serdang 43400, Selangor Darul Ehsan, Malaysia; sandra@upm.edu.my; 5 Genetics and Regenerative Medicine Research Centre, Faculty of Medicine & Health Sciences, Universiti Putra Malaysia, Serdang 43400, Selangor Darul Ehsan, Malaysia; 6 UPM-MAKNA Cancer Research Laboratory, Institute of Bioscience, Universiti Putra Malaysia, Serdang 43400, Selangor Darul Ehsan, Malaysia

**Keywords:** *Staphylococcus aureus*, MRSA colonization, neckties, headscarves, identification badges, medical students

## Abstract

Methicillin-resistant *Staphylococcus aureus* or MRSA infection is virulent and presents with a broad spectrum of severity. Limited regional reports that specifically outlined the potential risk of medical students being part of the dissemination of MRSA in healthcare settings were noted. This study aims to assess the prevalence and contributory factors of colonization of MRSA on neckties, headscarves, and ID badges among medical students in a local medical university in Malaysia. A cross-sectional study was conducted involving 256 medical students. A validated questionnaire was used to collect the data, and sample swabs were collected between July and August 2013 by swabbing neckties, headscarves, or identification badges. The swabs were then streaked onto mannitol salt agar (MSA) and incubated at 37 °C overnight. Out of 433 samples taken, 40 swabs (9.24%) were positive for *Staphylococcus aureus*. Out of the 40 swabs, five (12.5%) isolates were MRSA (one culture was isolated from the headscarf of a preclinical student, one culture was isolated from the necktie of clinical students, while the remaining three were isolated from identification badges of clinical students. There was no significant association between age, gender, ethnicity, and phase of medical students with the colonization of MRSA (*p* > 0.05). There was a significant association between knowledge score on hand hygiene practice and phase of medical students. MRSA colonies were present on neckties, headscarves, and identification badges of medical students of all phases. The findings from this study suggest the need for improvement of hand hygiene knowledge and discontinuity of mandatory use of physical ID badges and neckties among medical students.

## 1. Introduction

Hospital-associated methicillin-resistant *Staphylococcus aureus* (MRSA) is the most common cause of nosocomial infections and multidrug-resistant healthcare-associated infection [1]. Risk factors for MRSA mainly include immunosuppression, hemodialysis, extended hospital stays, and advanced age [2]. MRSA is a Gram-positive Staphylococcus strain that is resistant to widely used antibiotics known as betalactams such as methicillin, oxacillin, and penicillin. Generally, MRSA can be divided according to its etiology, namely, healthcare-associated and community-associated MRSA.

MRSA is highly prevalent in hospitals worldwide with the highest rates (>50%) reported in North and South America, Asia, and Malta [3]. The infection of MRSA in healthcare settings may lead to life-threatening conditions, which include meningitis, pneumonia, and infective endocarditis [4]. Contributory factors include compromised immune systems among inpatients that may worsen the disease, which can be acquired via healthcare instruments or casual contact from visitors or healthcare workers themselves [5]. MRSA has also been associated with surgical wound infections, urinary tract infections, bloodstream infections, and pneumonia [5].

The national prevalence rate of MRSA among *S. aureus* clinical isolates was 19.8% in 2017 [6]. The contributing factors to MRSA spread include poor health care worker hygiene, inadequate barrier nursing, antibiotic resistance, and an increase of potential number carriers and their fomite use. Items that are in contact with skin may serve as a fomite in MRSA transmission, which includes attire such as white coats and ties as well as stationery items such as pens [7]. With the rise in the sheer number of medical students in Malaysia, there appears to be a potential source of preventable threat to the spread of MRSA to patients. The medical students’ use of fomite and personal hygiene would need to be considered in prevention measures.

MRSA can be transmitted through direct hand contact with contaminated body fluid or contaminated stethoscopes [8], identification badges [9], neckties [10], and white coats [11], all worn by healthcare workers (mainly doctors and medical students) and it can also be transmitted through contaminated surfaces. Interestingly, a study on one of the medical universities showed that the presence of MRSA on neckties was only detected on doctors and not the medical students [12].

The objective of the study is to detect the colonization of MRSA on neckties, headscarves, and identification (ID) badges among medical students and to determine the association of MRSA colonization with socio-demographic characteristics and knowledge score on hand hygiene. We would also like to explore the role of medical student’s fomites as a possible source of MRSA-spread in a local health care setting.

## 2. Results

### 2.1. Distribution of Socio-Demographic Characteristics

In this study, the presence of MRSA colonies on medical students’ neckties, headscarves, and identification badges was detected using standard culture and PCR confirmation methods. Table 1 shows the distributions of the socio-demographic characteristics of the respondents.

### 2.2. The Colonization of Staphylococcus aureus and MRSA on Neckties, Headscarves, and ID Badges among Medical Students

Colonization of *S. aureus* from 433 samples that were collected from neckties, headscarves, and ID badges used by medical students showed 40 samples were positive for *S. aureus* colonization (Table 2). Out of the 113 male students, 112 (99.1%) wore neckties. The results showed that out of the 112 neckties samples swabs, only ten (8.9%) were positive for *S. aureus* colonization (one preclinical and nine clinical students). Out of the 143 female respondents, 100 (69.9%) wore headscarves. The results showed that out of the 100 headscarves samples swabs, only 12 (12.0%) were positive for *S. aureus* colonization. Out of the 256 respondents, 221 (86.3%) wore ID badges. The results showed that out of the 221 ID badges samples swabs, only 18 (8.14%) were positive for *S. aureus* colonization.

Table 3 shows that the prevalence of colonization of *S. aureus* on neckties was significantly (*p* = 0.005) higher in medical students in the clinical years as compared to students in the preclinical years. Interestingly, 80% of those who were positive for *S. aureus* admitted that they did not button up their white coats during lab practical or while examining patients. There was no significant association between the prevalence of colonization of *S. aureus* on neckties and age and ethnicity.

This study reports that 87% of clinical students have never cleaned or sanitized their ID badges. Table 4 shows that there is a significant (*p* < 0.05) association between the prevalence of colonization of *S. aureus* on ID badges and ethnicity. There is also a significant (*p* < 0.05) association between the prevalence of colonization of *S. aureus* on ID badges and phase of study (clinical/preclinical years).

Table 5 shows the prevalence of colonization of MRSA on neckties, headscarves, and ID badges among medical students. The results showed that out of the 112 neckties samples swabs, only one (from a clinical student) tested positive for MRSA, giving a prevalence 0.89%. A hundred headscarves samples were collected, with 12 students testing positive for *S. aureus*, and only one (1.0%) testing positive for MRSA. For ID badges, 221 samples were collected, and five preclinical students and 13 clinical students tested positive. From that, three (1.4%) clinical students were positive for MRSA. There was no significant association between colonization of MRSA on neckties, headscarves, and ID badges and age, gender, and study year of medical students.

### 2.3. Association of Knowledge Score and Hand Hygiene Practices with Socio-Demographic Characteristics of Medical Students

A significant association between knowledge score on hand hygiene practice and preclinical vs. clinical medical students was observed (Table 6). The result shows 146 (57%) out of 256 medical students had a good knowledge score on hand hygiene practices. Out of 103 clinical students, 69 (67%) of them had good knowledge of hand hygiene practices. The results showed that there is an association between knowledge score on hand hygiene practice and preclinical vs. clinical medical students, as the *p*-value is less than 0.05 (X^2^ = 6.975, df =1, *p* = 0.008).

## 3. Discussion

The results show medical students in their clinical years who wear neckties and ID badges are more likely to be contaminated with *S. aureus* compared to those worn by preclinical medical students who have minimal exposure to hospital settings. The MRSA on clinical students’ neckties and ID badges might be because of exposure to hospital settings as opposed to preclinical students who are not yet exposed to hospitals.

MRSA colonization has a higher prevalence in the neckties worn by clinical students. Out of the 10 (25.0%) neckties worn by clinical students that were positive for *S. aureus* (*p* = 0.005), 1 (10.0%) had MRSA isolates. Furthermore, unrestrained neckties have the potential to make contact with patients easily because of their tendency to swing freely as the wearer leans forward, and it is not machine-washable [13]. Studies have shown that 70% of doctors never cleaned their neckties, and the remainder reported their neckties are cleaned on average once in 20 weeks [14]. Meanwhile, in our study, 48% admitted to never washing their neckties, while the remaining 52% admitted to washing their neckties at least once a month.

For ID badges, 3 (6.7%) came back positive with MRSA from 18 (45.0%) of those who were positive for *Staphylococcus aureus*. The pendulous nature of ID badges that are attached to lanyards and hang around the front of the body may increase contact with patients and thus have higher chances of being colonized with MRSA [13]. Interestingly, the poor hygiene of the ID badges worn by clinical medical students was postulated to increase measure taken the chances of ID badges being colonized with the nosocomial pathogen.

To our knowledge, this is the first study to report MRSA colonization in headscarves. One preclinical student tested positive for MRSA. Twelve cultures were positive for *S. aureus* (30.0%), and from those, 1 (8.33%) was positive for MRSA and was from a preclinical student. In our study, out of 63 preclinical students, 13 (20.0%) admitted to washing their headscarves at least once every fortnightly. In contrast, only 8.0% of clinical students washed their headscarves more frequently, which is every fortnight, weekly, or daily.

Medical students in their clinical years have a higher percentage of having good knowledge of hand hygiene practice compared to preclinical students [15,16]. The result shows 146 (57%) out of 256 medical students have a good knowledge score on hand hygiene practices. Out of 103 clinical students, 69 (67%) of them had good knowledge, which is slightly higher compared to preclinical students (50.3%). This is supported by previous studies that have indicated that 80–90% of clinical students show the right level of knowledge and awareness regarding hand hygiene practices and its importance as they have practiced it more while working in a clinical environment [17,18]. There is a significant association between knowledge scores on hand hygiene practices and the study phase of medical students.

Physician in formal attire (i.e., white coat, necktie) has been reported to portray a professional image to patients and may favorably influence trust and confidence-building [19,20]. However, contradicting results were observed in the Australian study that reported even without wearing a necktie, patients’ confidence or satisfaction in doctors did not diminish as long as they are neatly attired [21]. Displaying ID badges are essential for healthcare as they serve as a secondary form of identification used to access controlled areas. Several studies have shown that cleaning or disinfecting ID badges is associated with the incidence of pathogen colonization [22,23]. The relevance of neckties and ID badges as a part of formal physician attire warrants further investigation as they have the potential to serve as a vector for nosocomial pathogen.

In retrospect, the number of clinical students as respondents should be increased to improve the validity of results. Further studies, including nasal carriage of *S. aureus,* should be performed to determine the transmission of *S. aureus* among medical students, patients, environment, and clothing. These strains should then be analyzed by molecular methods, e.g., Staphylococcal cassette chromosome mec (SCCmec) classification and typing methods, multilocus sequence typing (MLST), and pulsed-field gel electrophoresis (PFGE), which will further contribute to the determination of the transmission model of *S. aureus* among medical students. Health authorities should take precautions on dress codes like wearing neckties, headscarves, and identification badges among medical students and health care workers to reduce the potential risk of harboring and transmitting life-threatening *S. aureus* and MRSA. This is in line with the Malaysian Medical Association’s plea to discontinue the use of neckties as part of the hospital setup. Moreover, proper hand hygiene and attitude when in contact with patients should be adhered to in order to reduce the risk of transmitting *S. aureus* to others, especially immunocompromised patients [24].

This study was conducted at a single teaching center with 251 respondents. An increase in sample size may have yielded more statistically significant results. MRSA colonization was assessed in headscarves, neckties, and ID badges. It has been shown that MRSA colonization was also observed in other sites, such as whitecoats. Hence, this may lead to an underestimation of MRSA prevalence among clinical students that frequently use whitecoats compared to preclinical medical students.

## 4. Materials and Methods

### 4.1. Study Population and Sampling Criteria

The study was approved by the Medical Research and Ethics Committee, Faculty of Medicine and Health Science, Universiti Putra Malaysia. This cross-sectional study was conducted at the Faculty of Medicine and Health Sciences, Universiti Putra Malaysia (UPM), located in Serdang, Selangor Darul Ehsan, Malaysia. The sample size calculation was based on [25], which estimated 250 samples. A stratified proportionate to size sampling technique was used to select the respondents from each year of study. The sampling frame consisted of a list of students in each year of study. Out of the total 300 medical students, a total of 251 (83.7%) participated in the study. Those who did not wear neckties, headscarves, or ID badges and not present during the day of data collection were excluded from this study.

### 4.2. Data Collection

A self-administered questionnaire was used to collect the data. The validated questionnaire was previously tested for its reliability on 30 respondents. The questionnaire consists of three sections, which include socio-demographic characteristics of medical students, information associated with MRSA colonization among medical students, and 11 questions on the knowledge of hand hygiene practices among medical students.

Students’ knowledge regarding hand hygiene practices was calculated based on the WHO marking scheme [26]. Briefly, each correct answer was given one point, and an incorrect answer was given zero. The maximum score obtainable for the knowledge was 28. The scores were calculated and expressed in percentage, with an overall score of more than 75% considered as good and <50% taken as poor.

### 4.3. MRSA Colonization

Swab samples were collected by swabbing the neckties, headscarves, or ID badges of medical students. The swabs were then streaked onto mannitol salt agar (MSA) and incubated overnight at 37 °C. The MSA was then examined for any indication of a distinctive golden yellow halo, which signifies *Staphylococcus aureus*. DNA was extracted from the colonies and was subjected to PCR amplification, as previously described [27]. Briefly, PCR amplification was performed using *mecA*—specific primer pairs that indicate the presence of MRSA. The amplified products were visualized by electrophoresis in 2% agarose gels stained with ethidium bromide.

### 4.4. Data Analysis

All data were analyzed using the Social Package for Social Science Version 21.0 (SPSS). For descriptive analysis, frequency and percentage were used to describe the categorical data. The association between variables of categorical data was determined using the chi-square test and Fischer’s exact test. The confidence interval was set as 95%, and the standard *p*-value of *p* < 0.05 was set for all significant levels.

## 5. Conclusions

Based on our findings, there was no significant association between age, gender, ethnicity, and study phase of medical students to present colonization of MRSA on neckties, headscarves, and ID badges. The presence of MRSA colonies was noted on neckties, headscarves, and ID badges among medical students. There was a significant association between the preclinical vs. clinical medical students and colonization of *S. aureus* on neckties and ID badges. A significant association was also noted between ethnicity and colonization of *S. aureus* on identification badges. There was a significant association of knowledge scores on hand hygiene practices and preclinical vs. clinical medical students.

## Figures and Tables

**Table 1:** Socio-demographic characteristics of respondents (*n* = 256).

Factors	N	Percentage (%)
Age		
19–20	65	25.4
21–25	191	74.6
Gender		
Male	113	44.1
Female	143	55.9
Race		
Malay	156	60.93
Chinese	75	29.31
Indian	18	7.03
Others	7	2.73
Current Year		
1st	66	25.8
2nd	87	34.0
4th	62	24.2
5th	41	16.0

**Table 2:** Colonization of *S. aureus* on neckties, headscarves, and ID badges (*n* = 433).

Attires	The Colonization of *Staphylococcus aureus*		
	Positive, *n* (%)	Negative, *n* (%)	Total, *n*
Neckties	10 (8.9)	102 (91.1)	112
Headscarves	12 (12)	88 (88)	100
ID Badges	18 (8.14)	203 (91.86)	221
Total	40 (9.24)	393 (90.76)	433

**Table 3:** Association of the socio-demographic characteristics of medical students with the colonization of *S. aureus* on neckties.

Characteristics of Medical Students	The Colonization of *Staphylococcus aureus* on Neckties			Chi-SquareTest Value		
	Positive, *n* (%)	Negative, *n* (%)	Total	X^2^	Df	*p*-Value
Overall	10 (8.9)	102 (91.1)	112			
Age						
16–20	1 (3.2)	30 (96.8)	31			
21–25	9 (11.1)	72 (88.9)	81	1.714	1	0.279
Ethnicity						
Malay	4 (7.1)	52 (92.9)	56			
Non-Malay	6 (10.7)	50 (89.3)	56	0.439	1	0.508
Medic phase						
Preclinical	1 (1.6)	60 (98.4)	61			
Clinical	9 (17.7)	42 (82.3)	51	8.753	1	0.005 *

* X^2^ significant at *p* < 0.05.

**Table 4:** Association of the socio-demographic characteristics of medical students with the colonization of *S. aureus* colonies on ID badges.

Characteristics of Medical Students	Colonization of *S*. *aureus* on ID Badges			Chi-Square Test Value		
	Positive, *n* (%)	Negative, *n* (%)	Total	X^2^	Df	*p*-Value
Overall	18 (100.0)	203 (100.0)	221			
Age						
16–20	3 (5.45)	52 (94.55)	55			
21–25	15 (9.0)	151 (91.0)	166	0.708	1	0.572
Gender						
Male	6 (6.52)	86 (93.48)	92			
Female	12 (9.30)	117 (90.7)	129	0.555	1	0.456
Ethnicity						
Malay	15 (11.45)	116 (88.55)	131			
Non-Malay	3 (3.33)	87 (96.67)	90	4.698	1	0.030 *
Medic phase						
Preclinical	5 (4.06)	118 (95.94)	123			
Clinical	13 (13.27)	85 (86.73)	98	6.171	1	0.013 *

* X^2^ significant at *p* < 0.05.

**Table 5:** Colonization of MRSA on neckties, headscarves, and ID badges among medical students.

Attires	Colonization of MRSA		
	Positive, *n* (%)	Negative, *n* (%)	Total, *n* (%)
Neckties	1 (0.89)	111 (99.11)	112
Headscarves	1 (1)	99 (99)	100
ID Badges	3 (1.36)	218 (98.64)	221
Total	5	428	433

**Table 6:** Association of knowledge score on hand hygiene practices and preclinical vs. clinical medical students.

Phases of Medical Students	Knowledge Score			Chi-Square Test Value		
	Poor Knowledge, *n* (%)	Good Knowledge, *n* (%)	Overall, *n* (%)	X^2^	Df	*p*-Value
Medical student						
Preclinical	76 (49.7)	77 (50.3)	153 (100.0)			
Clinical	34 (33.0)	69 (67.0)	103 (100.0)	6.9	1	0.008

* X^2^ significant at *p* < 0.05.
